# At the nucleus of cancer: how the nuclear envelope controls tumor progression

**DOI:** 10.1002/mco2.70073

**Published:** 2025-01-24

**Authors:** Francesca Paganelli, Alessandro Poli, Serena Truocchio, Alberto M. Martelli, Carla Palumbo, Giovanna Lattanzi, Francesca Chiarini

**Affiliations:** ^1^ Department of Biomedical and Neuromotor Sciences Alma Mater Studiorum University of Bologna Bologna Italy; ^2^ IFOM ETS ‐ The AIRC Institute of Molecular Oncology Milan Italy; ^3^ Department of Biomedical Metabolic and Neural Sciences University of Modena and Reggio Emilia Modena Italy; ^4^ CNR Institute of Molecular Genetics “Luigi Luca Cavalli‐Sforza” Unit of Bologna Bologna Italy; ^5^ IRCCS Istituto Ortopedico Rizzoli Bologna Italy

**Keywords:** cancer, chromatin remodeling, lamins, mechanotransduction, nuclear envelope

## Abstract

Historically considered downstream effects of tumorigenesis—arising from changes in DNA content or chromatin organization—nuclear alterations have long been seen as mere prognostic markers within a genome‐centric model of cancer. However, recent findings have placed the nuclear envelope (NE) at the forefront of tumor progression, highlighting its active role in mediating cellular responses to mechanical forces. Despite significant progress, the precise interplay between NE components and cancer progression remains under debate. In this review, we provide a comprehensive and up‐to‐date overview of how changes in NE composition affect nuclear mechanics and facilitate malignant transformation, grounded in the latest molecular and functional studies. We also review recent research that uses advanced technologies, including artificial intelligence, to predict malignancy risk and treatment outcomes by analyzing nuclear morphology. Finally, we discuss how progress in understanding nuclear mechanics has paved the way for mechanotherapy—a promising cancer treatment approach that exploits the mechanical differences between cancerous and healthy cells. Shifting the perspective on NE alterations from mere diagnostic markers to potential therapeutic targets, this review calls for further investigation into the evolving role of the NE in cancer, highlighting the potential for innovative strategies to transform conventional cancer therapies.

## Graphical abstract

The review is focused on exploring novel intriguing perspectives on the role of the nuclear envelope in tumor progression. Alterations of nuclear envelope proteins result in the dysregulation of cellular pathways and promote tumorigenesis, highlighting that complex regulatory mechanisms lie behind the alterations in nuclear shape in cancer, thus revealing that these mechanisms could potentially open new frontiers for cancer therapies.

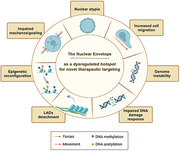

## 1 INTRODUCTION

**FIGURE 1:**
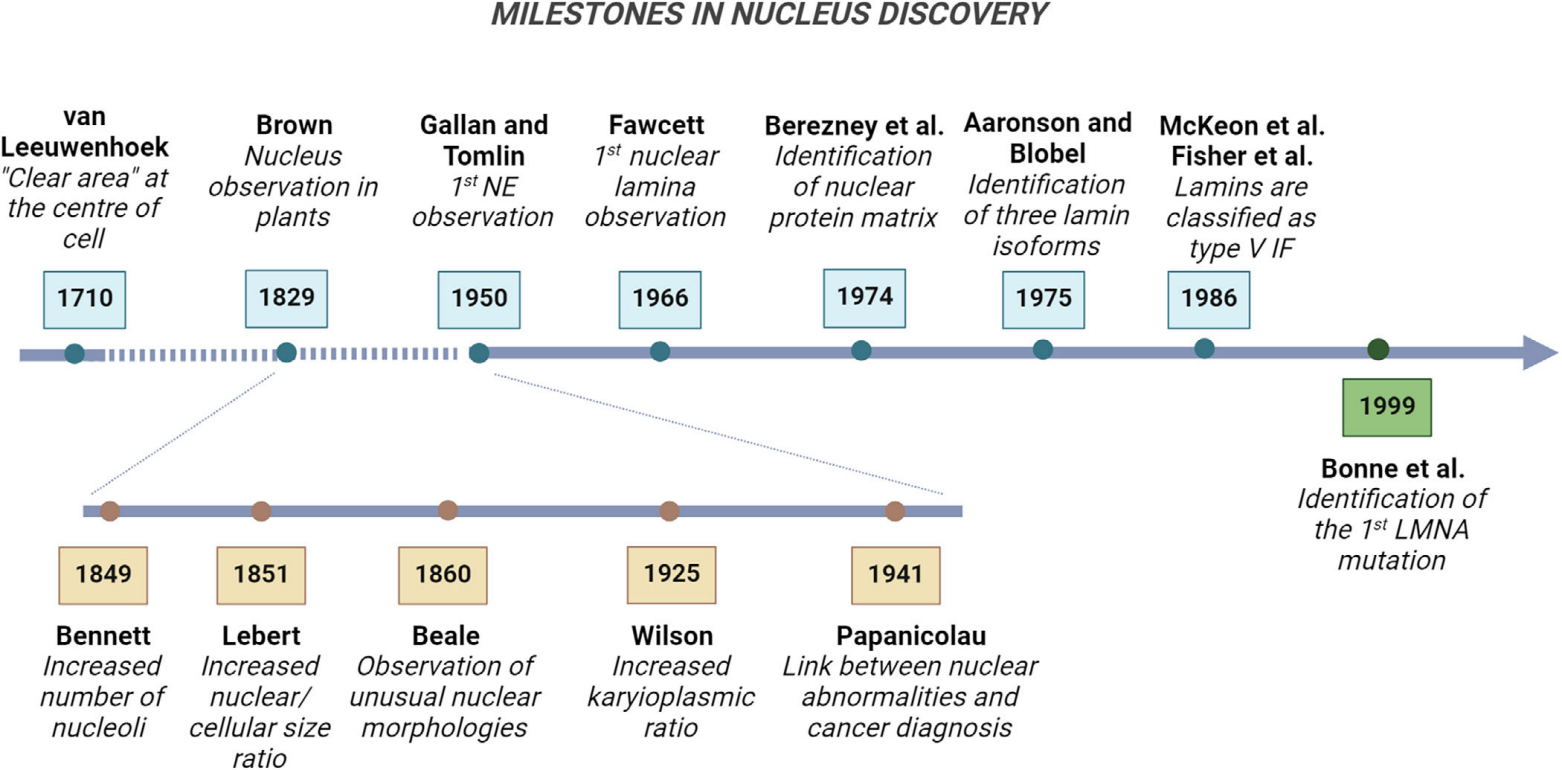
Timeline of key milestones in the understanding of the composition and organization of the cell nucleus in healthy (light blue boxes), cancer (light brown boxes), and laminopathic (green box) cells. Created in BioRender.com. IF, intermediate filaments; *LMNA*, lamin A gene; NE, nuclear envelope.

It was the early 1700s when Anthony van Leeuwenhoek, often hailed as the father of optical microscopy, peered through his lens to reveal a central “clear area” within amphibian and avian erythrocytes.^1^ Building upon this groundwork, a century later, botanist Robert Brown introduced the concept of nucleated cells as fundamental structural units in plants and coined a term that would last in biology books to this day: the *nucleus* ^2^ (Figure 1). Although initially meant only to denote its central position within the cell, over the centuries, the nucleus lived up to its name and earned the title of the core element of the cell theory. Indeed, acting as a physical barrier for genome containment and macromolecule diffusion, its enveloping membrane safeguards cell functions. Yet, recent advancements in cancer research have unveiled a more intricate narrative. In the realm of tumor biology, traditional studies have revolved around a genomic‐centric view, dating back to the discovery of oncogenes and tumor‐suppressor genes and the idea that multiple mutations are prerequisites for cancer initiation and progression.^3^ Over the past two decades, however, structural components of the nucleus—once seen as mere DNA shields—have emerged as highly dynamic elements actively orchestrating all phases of cancer development.

The existence of distinctive changes in the size and shape of cancer cell nuclei are anything but recent discoveries, having served as significant diagnostic indicators for centuries.^4^, ^5^ While healthy cells typically display nuclei with an ellipsoidal form and smooth outlines, malignant cells often have enlarged nuclei with irregular contours and altered chromatin organization. In the mid‐nineteenth century, the British physician Lionel Smith Beale attributed diagnostic significance to nuclear size and shape variations.^5^ Early work by another English physician, John Hughes Bennett, also revealed the presence of multinucleation and increased nucleolar counts in cancer cells^5^ (Figure 1). The French/German pathologist Hermann Lebert then reported a distinct, elevated ratio between nuclear and cellular size—later termed karyoplasmic ratio—in malignant cells.^6^ Since then, nuclear morphological criteria have become commonplace in cytopathology labs, helping to differentiate between benign and malignant cells, streamlining diagnosis, and offering prognostic and management guidance for decades.^6^, ^7^ In this regard, seminal is the work of George Papanicolaou's *Diagnosis of Uterine Cancer by the Vaginal Smear*, laying the foundation for the widespread cervical screening “pap test.”^6^ Subsequent efforts to advance the field have aimed to assess cancerous nuclei by delving into the mechanisms underlying their morphological changes. Some of the mentioned alterations could stem from cancer‐related changes in DNA content or chromatin organization.^8^, ^9^ However, it was not until five decades after electron microscopy first revealed them in the 1940s, that the main components of the nuclear envelope (NE) also started being recognized as crucial factors in cancer progression^2^ (Table 1).

**TABLE 1:** Key landmarks in understanding the structural and functional role of the NE in normal or pathological conditions.

Date	Type of discovery/progress	References
1710	Description of a “clear area” within amphibian and avian erythrocytes	^1^
1832	Introduction of the term “nucleus” observed in several plants	^2^
1849	Description of multinucleation and increased nucleolar counts in cancer cells	^5^
1851	Observation of elevated ratio between nuclear and cellular size in cancer cells	^6^
1860	Description of unusual nuclear morphology in cancer cells	^6^
1925	Observation of increased karyoplasmic ratio in cancer cells	^6^
1941	Establishment of a linking between nuclear abnormalities and cancer diagnosis	^6^
1950	1st observation of the NE and discovery of large pores within the envelope	^2^
1952	Recognition of two distinct layers within NE structure	^2^
1959	Pioneering studies in the automation of diagnostic parameters	^5^
1964	Standardization of morphometric parameters for cancer diagnosis	^5^
1966	Discovery of the nuclear lamina	^2^
1967	Identification of the octagonal structure of nuclear pores	^10^
1974	Identification of nuclear protein matrix	^11^
1975	Discovery of the three lamin isoforms	^12^
1986	Classification of lamins as intermediate filaments	^13^, ^14^
1994	Discovery of emerin and its mutations in Emery–Dreifuss muscular dystrophy	^15^
1999	Identification of the first *LMNA* mutation	^16^
2000	Identification of 41 *LMNA* mutations resulting in laminopathies	^17^
2002	Identification of the first *LMNA* mutation linked to accelerated aging	^18^
2003	Discovery of 67 novel nuclear membrane proteins with potential disease implications	^19^
2009	Creation of emerin‐based nucleus 3D reconstruction for cancer diagnosis	^20^
2017	Recognition of the markedly different structure of lamins compared with cytoplasmic intermediate filaments	^21^

Abbreviations: *LMNA*, lamin A gene; NE, nuclear envelope.

**FIGURE 2:**
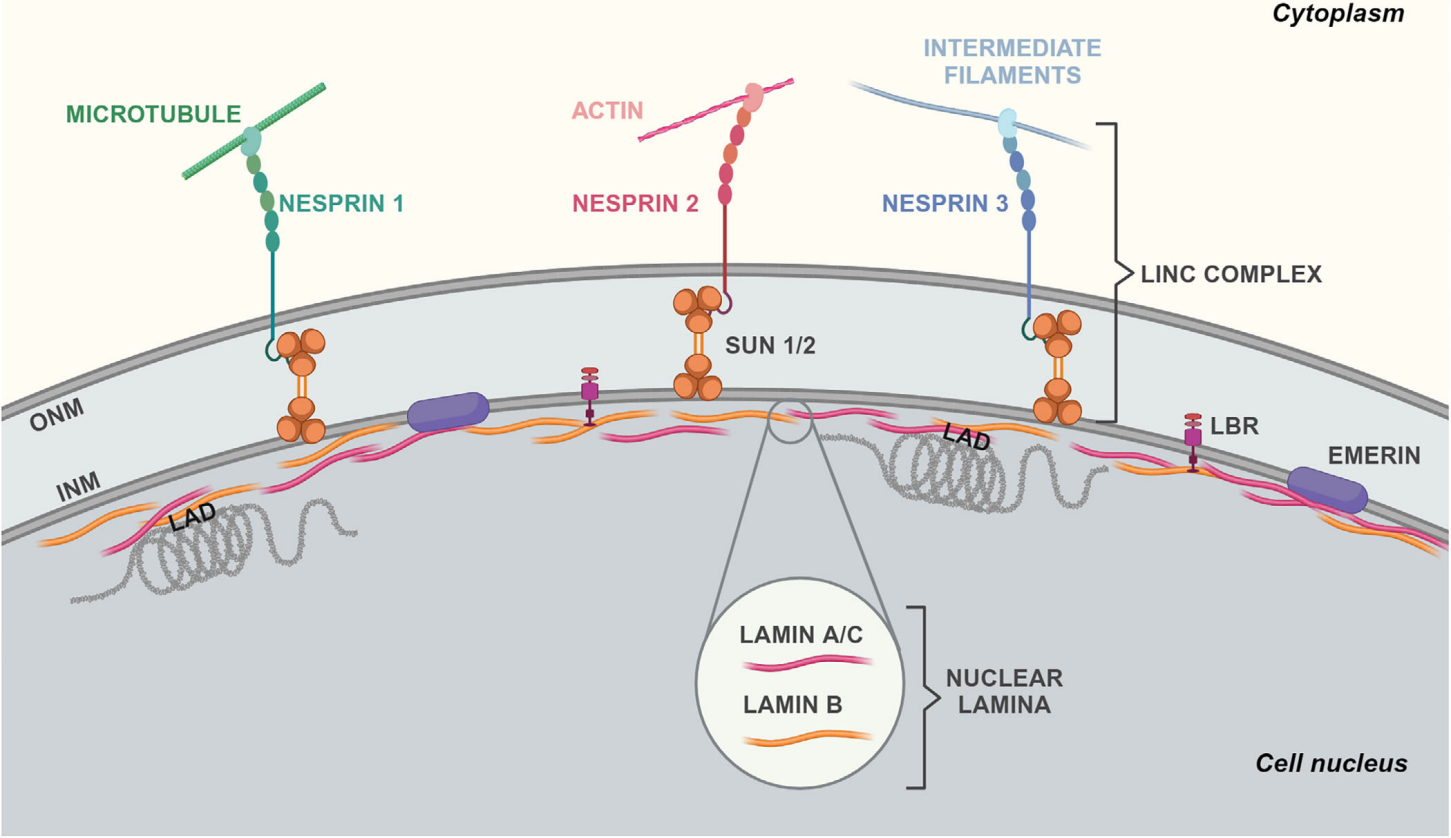
Schematic depiction of cell nucleus organization in healthy cells. The NE comprises the INM and the ONM. The LINC complex contains SUN and nesprin proteins. Nesprin proteins connect the nucleus to the cytoskeleton, while INM proteins interact with the nuclear lamina composed of lamin A/C and lamin B. Lamin monomers consist of an N‐terminal head domain, a coiled‐coil central rod domain, and a globular C‐terminal domain containing an Ig‐like fold. Tetramers of lamins assemble into filaments with a thickness of 3.5–7 nm that, in turn, are woven into a 30–100‐nm‐thick meshwork. The nuclear lamina interacts with heterochromatic regions known as LADs. LEM‐domain containing protein emerin regulates mechanotransduction by binding to lamins. Created in BioRender.com. INM, inner nuclear membrane; LAD, lamina‐associated domain; LBR, lamin B receptor; LINC, linker of nucleoskeleton and cytoskeleton complex; Ig, immunoglobulin; ONM, outer nuclear membrane; SUN‐1/2, Sad1p/UNC‐84 1/2.

The NE comprises two layers of lipid membranes separated by a luminal space, various embedded and membrane‐associated proteins, and a dense polymer network beneath its inner membrane, known as the nuclear lamina (Figure 2). The latter consists of the intermediate filament proteins called lamins and behaves as a load‐bearing shell that structurally supports the nucleus.^22^, ^23^ Starting with the discovery of emerin, in 1994, and lamin A/C, the main constituent of the lamina, in 1999, as genetic determinants of Emery–Dreifuss muscular dystrophy, hundreds of mutations in *LMNA* and other genes encoding NE proteins emerged as the cause of various genetic diseases collectively known as laminopathies.^15^, ^16^, ^17^ Upon noticing a striking resemblance between aberrant nuclei of cancer and laminopathic cells,^24^, ^25^ scientists began to question whether NE alterations could also play a role in malignant transformation.^1^, ^26^ This concept was then increasingly bolstered by a growing body of literature detailing changes in the expression of lamins across different human tumors, frequently linked with particularly aggressive phenotypes. Beyond lamins, other NE proteins have recently shown a potential involvement in malignant transformations.

A genome‐wide investigation including individuals with breast, colorectal, or ovarian cancer, alongside a comprehensive study on a group of 100 breast cancer patients, identified mutations in NE proteins known as nesprins.^27^, ^28^ Expression levels and mutations in the same proteins correlated also to an increased susceptibility to invasive ovarian cancer.^29^ Changes in several proteins associated with the nucleoskeleton have been linked to aggressive tumor phenotypes and serve as prognostic markers.^30^, ^31^ Research on laminopathies has uncovered another aspect of NE proteins crucial for their role in cancer progression: the regulation of nuclear mechanical properties. Over the last decades, the mechanical characteristics of cancer cells have emerged as a distinctive feature influencing the growth, invasion, metastasis, and response to therapies of solid tumors.^32^

Once viewed as a passive player in cancer biology, the NE is now recognized as an active contributor to tumor progression, affecting cellular responses to mechanical forces. This shift in understanding highlights the urgent need to consolidate and interpret the expanding body of research on the NE's functional roles in cancer. This review offers a thorough analysis of the NE in cancer, exploring its evolution from a structural component to a crucial factor in tumor progression. We examine how NE components drive cancer cell mechanics, epigenetic reprogramming, cell growth, and metastasis. Moreover, we discuss cutting‐edge technologies—such as AI (artificial intelligence) for prognosis, mechanotherapy, and precision measurement tools—that hold great potential for advancing diagnostic and prognostic methods, with the promise to transform cancer treatment.

## 2 THE NE UNDER PHYSIOLOGICAL CONDITIONS

NE components are not just structural elements; they actively sense mechanical signals through interactions with the cytoskeleton, playing a key role in mechanotransduction. Additionally, NE proteins interact with specific chromatin domains, impacting gene regulation and epigenetic reprogramming. The following sections will analyze these crucial functions.

### 2.1 Structure and mechanics of the NE

The NE demarcates the nuclear interior from the cytoplasm and comprises two phospholipid bilayers, the inner nuclear membrane (INM) and the outer nuclear membrane (ONM), separated by a luminal space of 30–50 nm (Figure 2). Both INM and ONM can create extensive invaginations into the nucleoplasm, forming structures known as the nucleoplasmic reticulum,^33^, ^34^ with the ONM seamlessly connected to the rough endoplasmic reticulum (ER). Mechanically speaking, when the nucleus experiences osmotic swelling or mechanical compression, fluctuations in the NE area and nuclear invaginations diminish, resulting in a smooth surface. These morphological changes often indicate nuclear membrane (NM) tension,^35^, ^36^ which could partially relax upon structural changes in its associated lipid bilayers, proteins, or chromatin. Proteins redistribution among the cytoplasm, nucleoplasm, and NM upon force‐induced structural changes of nuclear components is characteristic of mechanotransduction.

Under physiological conditions, cells maintain their mechanical equilibrium via mechanotransduction, meaning the capacity to sense physical stimuli from the environment and convert them into biochemical signals. Force sensing often involves multiple cellular compartments, with the structure and composition of the NE playing crucial roles.^37^, ^38^, ^39^, ^40^ Although the nucleus stands out as the most rigid cellular structure, its viscoelastic properties allow it to deform significantly under external compressive or tensile forces.^1^, ^41^, ^42^ The nucleus can translate mechanical cues (e.g., substrate stiffness or adhesion strength) into changes in gene expression, which help regulate its mechanical properties. This ability depends on the physical connection between the surrounding cytoskeleton and certain components of the NE.^43^, ^44^ Research on these nuclear molecules has traditionally focused on their roles during mitosis and meiosis. Lately, however, attention has shifted to their functions in interphase cells and how they are altered in pathologies like cancer.^45^ Importantly, the nucleus can also carry out direct mechanotransduction, acting as an alternative sensory surface that directly detects external physical forces without relying on input from the cytoplasmic actomyosin (e.g., upon nuclear compression or stretching).^35^, ^46^, ^47^

At the cellular level, the distribution of NE proteins results in different forms of mechanotransduction, spanning from protein binding to the NM, transcription factors (TFs) redistribution between the cytoplasm and the nucleus, and transcriptional activation or repression. ONM and INM contain a distinguished set of tissue‐specific proteomes that orchestrate different cellular processes, ranging from bidirectional nucleoplasmic transportation and maintenance of nuclear architecture to chromatin organization and mechanotransduction.^48^, ^49^, ^50^ These over 250 proteins are either membrane‐embedded or peripherally attached.

The most complex macromolecular assemblies of the NE are the nuclear pore complexes (NPCs), responsible for regulating the transport of macromolecules in and out of the nucleus. Another multiprotein assembly that spans both INM and ONM is the linker of the nucleoskeleton to the cytoskeleton (LINC) complex. The LINC complex's fundamental structure comprehends SUN (Sad1p/UNC‐84) domain‐containing proteins spanning the INM and KASH (Klarsicht/ANC‐1/Syne homology) domain‐containing proteins spanning the ONM (Figure 2). At the nucleoplasmic front, different isoforms of SUN proteins bind to the NPC, lamins, and chromatin. In the lumen, SUN proteins engage with KASH‐domain proteins.^51^ In turn, different KASH‐domain‐containing nesprins directly and indirectly connect to filamentous actin (F‐actin) (nesprin‐1/2), microtubules (nesprin‐3/4), or intermediate filaments (nesprin‐3) in the cytoplasm^1^ (Figure 2). Pioneering research by the Ingber group in the 1990s supported the idea of forces being transmitted from the cell surface to the nucleus via the cytoskeleton.^52^ Subsequent studies identified the LINC complex as the physical link between the cytoskeleton and nucleoskeleton, serving as the conduit for these forces across the NE.^53^, ^54^ Mechanical changes in the nucleus, cytoskeleton, and extracellular matrix (ECM) are then interconnected, with alterations in the ECM stiffness affecting cytoskeletal organization and lamin A/C expression. This results in cells finely tuned to their physical surroundings. So much so that LINC complex disruption perturbs perinuclear actin and causes nuclear shape changes and disease phenotypes that, in part, resemble *LMNA*‐linked defects.^55^, ^56^

In addition to SUN proteins, other 70–100 proteins localize at the INM,^5^ including the LEM‐domain containing protein emerin (Figure 2). Emerin can regulate nuclear mechanics and mechanotransduction by binding to lamins, nesprins, nuclear actin, chromatin, and gene regulators.^57^ Emerin‐deficient or ‐mutant cell nuclei display abnormal shapes, mechanical stimuli responses, and gene transcription.^58^, ^59^ The underlying mechanosensing mechanisms likely involve emerin's redistribution within the NM, alongside its ability to control actin polymerization.^60^ Lamina and chromatin anchoring via its LEM domain may promote its retention on the INM and turnover.^61^ Additionally, nuclear deformation may conceal emerin‐binding sites on lamin A/C, and strain‐induced chromatin remodeling or phosphorylation of emerin may influence its interaction with the DNA‐binding protein barrier‐to‐autointegration factor (BAF).^62^, ^63^, ^64^, ^65^ Following mechanical stress or lamin A/C deficiency, emerin NM redistribution either enhances or hinders cytoplasmic actin polymerization.^66^, ^67^ This polymerization diminishes nuclear globular actin (G‐actin), which functions as a transcriptional coactivator and nuclear export factor.^68^ By regulating nuclear G‐actin, strain‐induced emerin redistribution can thus modulate the shuttling of transcription factors and gene expression.

Underneath the INM, the NE finds support from the nuclear lamina, a dense protein network primarily composed of filament V‐type lamins^50^ (Figure 2). Vertebrate lamins consist of two types, A and B. The former, lamin A and C, derive from alternative splicing of the *LMNA* gene, while the latter, lamin B1 and B2, are encoded by two genes, *LMNB1* and *LMNB2*, respectively.^69^ Lamin filaments exhibit exceptional resilience, absorbing force through structural changes. They undergo reversible deformation under low loads (<500 pN), stiffen under applied force, and break at loads exceeding 2 nN.^46^, ^70^ But not all lamins seem to play the same mechanistic role. Lamin A/C contributes to the reinforcement of the nuclear lamina, whereas cells lacking lamin B1 exhibit normal nuclear stiffness yet are prone to membrane instability and blebbing.^71^ The expression level, phosphorylation, and turnover of lamin A/C are linked to ECM stiffness and cytoskeletal tension, indicating a mechanochemical feedback loop between the lamina and actomyosin.^72^, ^73^, ^74^ Decreased cytoskeletal tension, as seen on soft substrates, results in elevated phosphorylation, increased solubility, and degradation of lamin A/C. Contrariwise, increased cytoskeletal tension decreases lamin A/C phosphorylation and enhances its levels. Adjusting the stiffness of the lamina to match that of the ECM acts as a defense mechanism against excessive nuclear deformation.^46^ Lamins also play a role in genomic stability through their interaction with key molecules such as BRCA1 (breast cancer gene 1), 53BP1 (p53 binding protein 1), and ATM (ataxia‐telangiectasia mutated).^75^, ^76^ Moreover, lamin A has been implicated in replication fork maintenance through its interaction with proliferating cell nuclear antigen.^77^ Thus, genome instability caused by replication stress could be another pathogenic effect of altered lamin A levels in cancer cells.

Accordingly, a defective nuclear lamina is associated with enhanced genome damage, impaired DNA repair mechanisms, and cellular death, characteristics observed across all laminopathies.^78^

### 2.2 Interaction between the NE and chromatin

**FIGURE 3:**
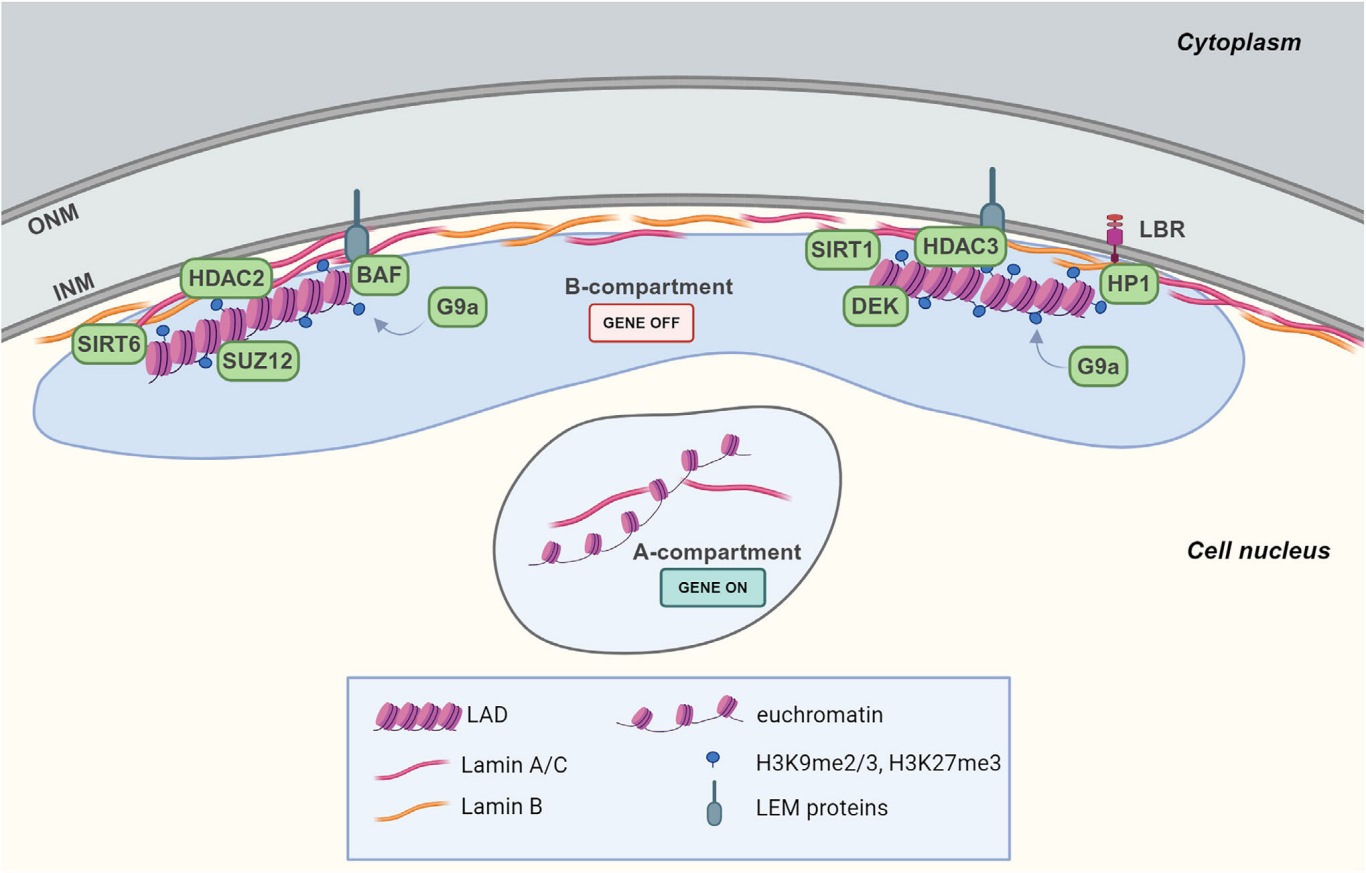
LADs tethering at the nuclear lamina. LADs are enriched for repressive heterochromatin marks (H3K9me2/3, H3K27me3). A schematic representation of proteins found to interact with either chromatin, histone deacetylase or methyltransferase as well as the nuclear lamina are reported. Proteins such as LEM proteins and LBR serve as molecular bridge or tether. LADs are localized in the B‐compartments, while euchromatin regions are situated in the nuclear interior (A‐compartments). Created in BioRender.com. BAF, barrier‐to‐autointegration factor; HP, heterochromatin protein; HDAC, histone deacetylase; INM, inner nuclear membrane; LAD, lamina‐associated domain; LEM, LAP2, emerin, MAN1; LBR, lamin B receptor; ONM, outer nuclear membrane; SIRT, sirtuin.

Lamins, emerin, and other lamina‐associated proteins directly and indirectly interact with the so‐called lamina‐associated domains (LADs) in the genome^79^, ^80^ (Figure 3). LADs typically span 10 kb to 10 Mb and feature reduced gene density and enhanced histone methylation marks typical of heterochromatin, such as histone H3 lysin 9 trimethylation (H3K9me3) and H3 lysin 27 trimethylation (H3K27me3).^81^ Some LAD‐nuclear lamina contacts are consistent across all cells, serving as a structural backbone, while others vary, exhibiting flexible interactions with the nuclear lamina.^82^

The positioning of LADs at the nuclear periphery is mediated by the chromatin state, and it is also dependent on lamins, INM proteins, chromatin readers and modifier proteins which directly interact with LADs or contribute to maintain the specific chromatin organization.^83^, ^84^

About the chromatin state, the presence of inactive chromatin markers (H3K9me2/3, H3K27me3) is required for LADs‐nuclear lamina association as the knockdown or inhibition of histone methyltransferases G9a, EZH2 (Enhancer of zeste homolog 2), SUV39H1, or SUV39H2, responsible for these methylations, resulted in weaker LADs‐nuclear lamina interactions in mammalian cells.^79^, ^85^, ^86^ The repression in LADs is also maintained by the interaction of lamin A with histone deacetylase (HDAC) 2 that promotes its deacetylase activity, and with the sirtuins SIRT1 and SIRT6.^87^, ^88^ Moreover, the association of emerin and thymopoietin/lamina‐associated polypeptide (LAP)2β proteins with HDAC3, also promotes histone deacetylation which reinforces gene silencing^89^, ^90^ (Figure 3). The importance of this repressive environment is confirmed by a recent study, conducted in mouse embryonic fibroblasts, that identified repressive chromatin modifier proteins, mainly involved in regulating the H3K9me2/3 methylation, as the more plentiful proteins enriched in LAD‐only interactome, such as HP (heterochromatin protein)1 family members, also known as chromobox (CBX) proteins. In particular, HP1α binds methylated H3K9, promoting gene silencing in LADs.^83^ Alongside these proteins, they also identified the histone chaperone DEK and the SUZ12 polycomb repressive complex 2 (PRC2) subunit which both enhance HP1 binding to H3K9me3, and the lysine methyltransferase G9a known to methylate H3K9 and H3K27 (Figure 3).^83^, ^91^, ^92^, ^93^

However, the chromatin state is not sufficient to tether LADs at the nuclear periphery, and the interaction with NE proteins is also necessary to stabilize peripheral heterochromatin (reviewed in Ref. ^94^).

In particular, lamin A tail contains a specific region which interacts with repressive modifications on the histone H3 contributing to heterochromatin tethering.^84^ The INM protein emerin is also involved in mediating a bridge between nuclear lamina and chromatin, through the physical interaction with lamin A.^90^

Moreover, BAF constrains LADs‐nuclear lamina connections binding to LEM‐proteins and histones.^90^ Lamin B receptor (LBR) also connects LADs to nuclear lamina by interacting with lamin B1 and HP1α which binds to the heterochromatic marker H3K9me3 (Figure 3).^84^, ^95^

Besides, the physical connection between NE components and chromatin yields two primary consequences on cell functionality. First, changes in the lamina‐chromatin contacts or deformations in the nucleus could alter gene distribution and activity. Second, chromatin structure alterations could, in turn, impact nuclear mechanical properties. DNA within the interphase nucleus is nonrandomly organized, with specific chromosomes occupying precise areas called chromosome territories.^96^ These territories have different levels of gene activity,^97^, ^98^ and nuclear deformation could alter their relative position. Chromosomes also intermingle, and these intermingling domains are directly linked with the transcriptional activity of genes within them.^99^, ^100^ The positioning of chromosome territories and their connection to the nuclear lamina through LADs regulate chromatin structure and transcriptional activity. Typically, chromatin‐nuclear lamina association correlates with transcriptional repression, as anchoring loci to the nuclear periphery results in gene silencing. The interactions between heterochromatin and lamina dictate the spatial segregation of stretches of active and inactive chromatin within the genome.^101^ The former, called A compartments (active euchromatin), are associated with nucleoplasmic lamin A and tend to reside in the nuclear interior, while the latter, termed B compartments (heterochromatin), are linked to the nuclear lamina^81^, ^102^, ^103^ (Figure 3). Since the nuclear lamina links chromatin to the extracellular environment, external force‐induced changes in gene expression could likely happen via alterations in chromatin topology, accessibility, and posttranslational modifications. These effects are cell type‐, force magnitude‐, and time frame specific. To give an instance, mechanical forces transmitted from cell adhesions to the nucleus caused rapid chromatin “stretching,” which was linked with the activation of mechanosensitive gene expression when applied continuously for 1 hour.^104^, ^105^ Transcription levels correlated with both frequency and magnitude of applied forces and relied on the connection to the LINC complex. This rapid transcriptional response suggested that chromatin stretching altered the transcriptional machinery accessibility to specific genes rather than modifying the epigenetic state of the locus. However, how forces mediate the activation of some genes but not others remains an open question. One hypothesis is gene positioning. The epidermal differentiation complex (*ECD*) gene cluster, for example, was inactive in undifferentiated epidermal stem cells, where it sat near the lamina. As stem cells differentiate, the locus relocated more centrally within the nucleus, correlating with *ECD* activation.^106^ On longer time scales, mechanical stimuli can also trigger active, biochemical signaling‐dependent changes in chromatin condensation. Thus, the cyclic straining of skin epidermis progenitor cells decreased H3K9me3 of gene‐poor peripheral heterochromatin. This caused higher heterochromatin mobility and nuclear softening.^63^, ^66^ Besides lamins, indeed, chromatin mechanical resistance governs the elastic deformations of the nucleus under small extensions (<3 µm).^107^ Mechanically speaking, the DNA coil exerts an outward entropic force on the nucleus by being physically constrained within its boundaries. DNA–protein interactions condense the DNA, which thus experiences an inward enthalpic force. Therefore, a change in chromatin status that favors heterochromatin enhances NM structural stability and nuclear stiffness, while the opposite leads to nuclear softening. Lamina alterations can also influence nuclear stiffness by affecting chromatin. Loss of lamins resulted in the separation of heterochromatin from the nuclear lamina and its dispersion across the nucleus, ultimately reducing nuclear stiffness.^108^, ^109^ Chromatin condensation status can also impact the actomyosin cytoskeleton and cellular mechanics. For example, reduced heterochromatin increased actomyosin cytoskeleton stiffness as a compensatory response in breast cancer cells, which may lead to increased cellular stiffness that reportedly impairs tumor cell migration ability.^110^, ^111^

Under physiological circumstances, the NE functions as a dynamic element that senses and responds to biomechanical and metabolic stimuli. Alterations in the equilibrium of its components, however, appear to underlie the development of numerous diseases, cancer included.

## 3 NE CHANGES IN CANCER PROGRESSION

Building on early findings regarding the involvement of NE proteins in tumorigenesis (summarized in Table 2), this section highlights recent studies on how different NE components influence and promote the malignant phenotype. This emerging area of research has yielded remarkable insights into the role of NE alterations in cellular transformation.

**TABLE 2:** Alterations of NE components in cancer.

Cancer type	Alterations of NE proteins	References
Breast cancer	Lower lamin A/C and lamin B1 expression; decreased levels of emerin in invasive breast cancer; downregulation of SUN1, SUN2, and nesprin‐2; upregulation of LBR.	^112^, ^113^, ^114^
Endometrial cancer	Lower lamin A expression	^115^
Small cell lung carcinoma	Lower lamin A/C and lamin B1 expression; downregulation of emerin	^116^, ^117^
Non‐small cell lung carcinoma	Higher lamin A/C and lamin B2 expression; downregulation of SUN2 in lung adenocarcinoma	^116^, ^118^, ^119^
Ovarian cancer	Lower lamin A expression; higher lamin B1 expression	^120^, ^121^
Gastric carcinoma	Lower lamin A/C expression	^122^
Colon cancer	Loss of lamin A/C in stage II and III; upregulation of lamin B2 in primary lesions; downregulation of SUN2	^123^, ^124^, ^125^
Prostate cancer	Higher lamin A/C expression	^126^, ^127^
Hepatocellular carcinoma	Higher lamin B1 expression	^128^
Myeloid neoplasms	Loss of lamin B1	^129^
Bladder cancer	Higher lamin B2 expression	^130^

This table represents a summary of dysregulated expression of NE components, in particular lamins, observed in several tumors. Here we reported the assessment of NE components in tumor samples versus their normal counterparts.

Abbreviations: LBR, lamin B receptor; NE, nuclear envelope; SUN, Sad1p/UNC‐84.

### 3.1 NE components and tumorigenesis

Both healthy and mutant cells exhibit a large array of nuclear shape variations as the cell adapts to external (e.g., migration through dense ECM) and internal (e.g., tensile forces from the actin cytoskeleton) constraints.^131^ For example, cancer cells experience significant changes in the mechanical cues within the tumor microenvironment (TME), such as ECM stiffness, confinement, stretching, and shear stress.^32^, ^132^, ^133^ These biomechanical stresses can activate signaling pathways that induce metabolic and behavioral changes in cancer cells, driving the acquisition of stem‐like properties and promoting cancer progression, metastasis, and genetic and epigenetic alterations. One proposed mechanism suggests that softer, more irregularly shaped nuclei may help cancer cells to invade densely packed tissues, where cells encounter constrictions narrower than the nuclear diameter.^134^, ^135^ Moreover, the metastatic process—spanning from primary tumor development to intravasation, extravasation, and the establishment of metastatic niches—is characterized by distinct mechanical properties, such as stretching from increased cancer cell proliferation and compressive stress from the TME.^136^

**FIGURE 4:**
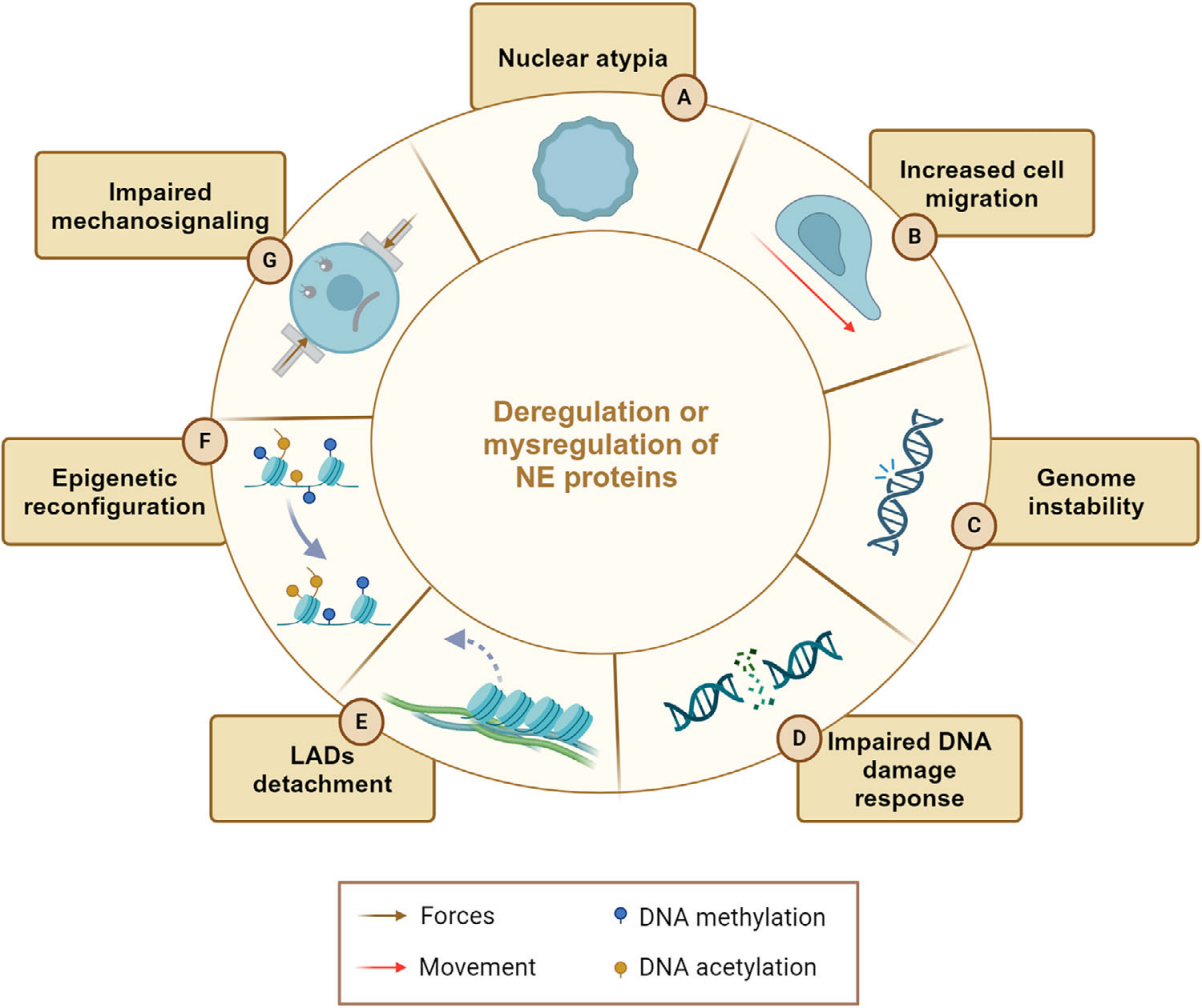
Overview of the main effects associated with NE protein alterations in cancer. Each light brown box indicates a mechanism linked to NE proteins deregulation resulting in tumor progression. (A) Low levels of lamins result in severe invagination of NE and nuclear blebs formation with loss of nuclear shape; (B) lamin A is required to maintain a correct NE composition and low lamin A levels contribute to decrease NE rigidity, thus enhancing cell migration; (C) NE plays a fundamental role in protecting the genome, and the loss of its integrity can allow uncontrolled content exchange between nucleus and cytoplasm which results in genomic instability of cancer cells; (D) NE proteins are involved in DNA repair mechanisms. SUN1/2 genes knockdown leads to accumulation of DNA damage with compromised DNA repair mechanisms; also, lamin A loss results in impaired DNA reparation; (E) changes in lamin levels and loss of NE proteins localization impair LADs tethering at the nuclear lamina with alterations in heterochromatin organization; (F) alterations of NE proteins can influence 3D chromatin organization with consequences in cancer epigenetic reprogramming; (G) mislocalization of NE proteins (i.e., lamins, LINC complex) impaired mechano‐signaling pathways, thus promoting a more aggressive cancer phenotype. Created in BioRender.com. LAD, lamina‐associated domain; NE, nuclear envelope.

Mechanical inputs often lead to NE ruptures (NER), able to originate genomic aberrances and alterations which might favor or disfavor cell growth, migration and resistance to anticancer‐therapy treatments. In this respect, invading tumor cells and circulating tumor cells (CTC) undergo pulling, pushing and compression events that trigger NER before reaching the bloodstream and disseminating in the human body.^22^, ^137^, ^138^, ^139^ On the other hand, genomic analyses showed CTC isolated from the same patients are characterized by deep genomic heterogeneity and chromosomal instability.^140^, ^141^ Altogether, these studies support the idea that mechanically‐driven NER might play a fundamental role in the establishment of genomic diversity, which is often correlated with cancer progression. Similarly, the composition of the NE influences both the flexibility and morphology of the nucleus and, in turn, the cellular response to mechanics. Mutations in these components can thus account for various nuclear shape alterations commonly seen in cancer. For example, many cancer types present with alterations in lamin A/C, which is downregulated in lymphoma, leukemia, osteosarcoma, breast, and ovarian cancer cells,^142^, ^143^, ^144^, ^145^ and upregulated in prostate cancer and glioblastoma multiforme.^126^, ^146^ Lamin A/C expression levels may vary within tumor subtypes as well. While non‐small cell lung carcinomas strongly express lamin A/C, in small‐cell lung ones, protein is either low or absent.^116^, ^142^ Alterations in LINC complex proteins occur in human tumors and frequently correlate with a more aggressive phenotype.^29^, ^147^, ^148^ Recently, our group reported that the mislocalization of emerin, SUN1, SUN2, and nesprin via a lamin A‐depending mechanism promotes a more aggressive phenotype in Ewing sarcoma.^149^ A global loss of NE proteins has been observed in human breast cancer tissues, suggesting that reduced expression of lamins and LINC complex proteins may have a role in the progression of this type of tumor.^112^ Overall, these studies unveil a deep connection between changes in NE proteins and pathological traits across various cancer types resulting in increased cell migration, genome instability, epigenetic reconfiguration, and impaired mechanosignaling^32^, ^150^ (Figure 4). As the NE is recognized as a central hub for various cellular processes, research is now increasingly focused on establishing a causal link between changes in NE proteins and cancer progression.

### 3.2 Lamin A and C

The precise nature of lamin A/C levels in cancer remains uncertain. They could represent a dynamic response to the TME and signaling pathways at a given time or an independent initiation of oncogenic changes decoupled from regulatory mechanisms. As mentioned above, both increased and decreased levels of lamin A/C correlated to poor prognosis in human cancers.^142^ These seemingly contradictory findings may stem from tissue‐specific functions, variability in expression within a single tumor, or dynamic changes in response to biochemical or mechanical signals during tumor progression. Specific oncogenic transformations can also lead to alterations in lamin expression that vary widely and may exhibit opposite effects. One mechanism of lamin A/C deregulation in hematological malignancies is the epigenetic silencing of *LMNA* gene by CpG island promoter hypermethylation which results in the loss of A‐type lamins expression in leukemias and lymphoma.^151^ Another study associates low lamin A levels with the overexpression of caspase 6 in ovarian cancer, thus linking the downmodulation of lamin A to its increased degradation.^152^ Alongside expression levels, the functionality of nuclear lamins depends on an array of posttranslational modifications that impact lamin activity, stability, and interaction with other proteins.^153^ Furthermore, alternative splicing of the *LMNA* gene controls the relative expression levels of lamin A and C, significantly contributing to tissue‐specific differences. In cancers affecting breast, colon, liver, lung, ovary, thyroid, and prostate, the ratio of lamin C to lamin A mRNA appeared elevated compared with normal tissues.^143^ Even more intriguing is the observation that, differently from laminopathies, mutations, deletions, or copy‐number changes in the *LMNA* gene are rare in most human cancers. These infrequent occurrences might depend on a selective pressure on cancer cells to ensure their ability to regulate lamin levels following the changing TME.^148^ This characteristic appears to provide different advantages to tumor cells, as discussed below.

Reduced or depleted levels of lamin A can have various effects on tumor cell behavior, with cell migration being a particularly well‐studied aspect. Cancer cell movement requires a certain degree of nuclear flexibility when navigating through dense ECMs and narrow pores during processes such as extravasation and transit through the bloodstream.^148^ A change in lamin expression resulting in decreased NE stiffness could thus be advantageous. Lower lamin A levels correlated to increased NE flexibility, greater invasiveness, and worse prognosis in breast cancer cells.^154^ Underlying the downregulation of lamin A in this context seemed to be the PI3K/Akt oncogenic pathway, known to promote lamin A degradation and to be hyperactivated in 60% of breast tumors.^155^, ^156^, ^157^ On the other hand, Akt2‐driven phosphorylation of lamin A upon TGF (transforming growth factor) β treatment contributes to nuclear deformations and genomic instability during epithelial‐to‐mesenchymal transition (EMT).^158^ The phosphorylation of lamin A/C at Ser22 by Akt1, has also been identified as a determinant factor in softening the nucleus of colon cancer cells, thus promoting their confined migration.^159^ Another recent study found a link between increased cell migration speed and heightened nuclear deformability upon ATM inhibition and subsequent reduction of lamin A levels.^160^ ATM, known for its DNA double‐strand break repair role, is often mutated in various human cancers.^161^ In breast adenocarcinoma and fibrosarcoma cell lines, ATM inhibition or deletion significantly decreased both *LMNA* gene expression and lamin A protein levels, resulting in more deformable nuclei with longer protrusions. In a non‐small cell lung carcinoma model, the stabilization of lamin A/C due to direct interaction with the type VI intermediate filament protein nestin, seemed to confer an advantage to cancer cells by protecting them from senescence. Nestin interaction prevented lamin A/C proteasomal degradation, thus avoiding lamina dispersion and subsequent tumor cell senescence.^162^ Lamin A also influences the inflammatory pathways. Altered processing of prelamin A has been linked to the NF‐kB (nuclear factor kappa‐light‐chain‐enhancer of activated B cells) pathway and a gamma‐interferon‐like response, with downstream activation of interleukin 6 and STAT1/3 signaling.^163^ As a whole, a low‐grade inflammatory condition triggered by altered lamin A posttranslational maturation is established, and may have a role in cancer progression. Increased ECM stiffness is a prominent characteristic of cancer biology often triggered by hypoxia within the tumor milieu.^164^ Changes in ECM stiffness have been linked to several phenomena that promote invasion and metastasis, including amplified EMT, augmented cellular contractions within the actin cytoskeleton, and weakened cell adhesion.^164^, ^165^, ^166^, ^167^ In breast cancers, stiffness is often higher at the tumor edge, marked by increased variability along the invasive front.^168^, ^169^ Lamin A levels also depend on the mechanical properties of the microenvironment (ME). A groundbreaking study from Discher's group reported that lamin A expression scales with tissue stiffness.^74^ This observation paved the road to a series of investigations that, in recent years, have tied the dysregulated expression of lamins to changes in tissue stiffness under various pathological conditions, including cancer.^170^ The mechanical properties of the environment seem to influence also where metastases happen. The so‐called seed and soil theory, proposed by Stephen Paget in 1889 and later revised by James Ewing in 1929, suggests that the dissemination of cancer cells to distant sites is guided by the anatomical characteristics of the vascular system, determining the location of metastasis.^171^ This theory, still widely accepted in the scientific community, now conceptualizes *seed* as progenitor cells, while *soil* refers to the various components of the ME within the metastatized organ. The intrinsic expression of lamins in highly aggressive cancer cells could influence metastatic tropism. Recent findings reported an inverse correlation between the aggressiveness of pediatric osteosarcoma‐derived cell lines and lamin A/C expression levels. Specifically, the highly metastatic cell line 143B exhibits low levels of lamin A/C, contrary to the usual high expression observed in bone‐derived cells.^172^ These metastatic cells possess a lamin A/C:lamin B ratio more indicative of soft tissue rather than bone, aligning with their propensity to metastasize to the lungs. In other words, oncogenic transformations in cells alter the abundance ratio of lamin A/C to lamin B, leading to a mechano‐incompatibility of the cancer cells in the primary tumor ME. Restoration of lamin A/C expression via plasmid transfection reversed this behavior.^173^ Additional investigations showed that low aggressiveness of MCF‐7 breast cancer cells correlated with low levels of lamin A/C. In contrast, the highly metastatic MDA‐MB‐231 cells presented increased expression of lamin A/C, better suited to the properties of bone tissue, where they preferentially metastatize.^174^, ^175^ During metastasis, the growth at the secondary site is believed to be the critical step in the metastatic cascade and might necessitate a transition from an EMT to a mesenchymal‐to‐epithelial phenotype.^176^ Therefore, phenotypic adaptability could be the primary driver of tumor progression, closely linked to alterations in lamin levels and nuclear organization. In this context, increased rigidity may facilitate invasion and escape from the primary tumor. On the contrary, a softer ME at the secondary site may lead to decreased lamin A/C levels, supporting the enhanced plasticity associated with stem cells necessary for outgrowth and metastasis.^148^

As aforementioned, lower levels of lamin A/C may self‐select during invasion due to increased nuclear deformability that confers a selective advantage to migrating cells. At the same time, however, softer nuclei are more susceptible to the substantial deformation they undergo during cell migration, ultimately increasing their risk of NE rupture and DNA damage.^22^, ^177^, ^178^ In this respect, microfluidic experiments showed that low levels of lamin A/C result in decreased resistance to fluid shear stress by tumor cells.^179^ Recent investigations have unveiled that natural occurrences of NE ruptures exist within living organisms. They happen, for instance, in the developing hearts of embryos, where actomyosin‐driven nuclear rupture leads to the misplacement of DNA repair factors within the cytoplasm and results in excessive DNA damage.^180^ Furthermore, mutations in the *LMNA* gene can precipitate NE ruptures in skeletal muscle cells due to the forces exerted by the microtubule cytoskeleton.^78^, ^181^ In healthy tissues, NE ruptures and the uncontrolled exchange of material between the nucleus and cytoplasm often correlate with processes of senescence and aging, which affect cell proliferation, viability, and tissue functionality.^22^, ^78^ ^,^ ^138^, ^182^ Within tumor cells lacking the senescence checkpoint, this could result in abnormal invasiveness without halting proliferation and further genomic instability that promotes cancer progression.^139^ However, how mechanically induced NE ruptures damage the DNA remains poorly understood. Recently, a study shed some light on a possible mechanism in human breast cancer cells.^139^ In the mammary duct, cellular crowding seemed to lead to NE ruptures that allowed the ER‐associated exonuclease TREX1 (three prime repair exonuclease 1) to relocate to the postrupture nucleus, where it caused DNA damage. Notably, markers of DNA damage and NE rupture were concentrated at the invasive edge of human tumors, marked by higher stiffness.^168^, ^169^, ^183^ The same study also analyzed the association between TREX1 expression levels and overall survival in breast cancer patients using publicly available datasets. The analysis revealed a significant association between high TREX1 expression and reduced survival probability. However, further experiments are required to assess if these findings apply to other tumors. Indeed, in the MDA‐MB‐231 cell line, DNA damage upon confinement appeared to be both TREX1‐ and NE‐independent.^139^, ^184^ While there are cases in which mechanical stress‐induced DNA damage occurs in the absence of NE rupture,^22^, ^177^ the mechanisms behind it remain unclear. A recent study showed that the nuclear deformation resulting from confined migration or external compression of cancer cell lines could damage the DNA by increasing replication stress (likely due to heightened torsional stress in the DNA).^184^ The susceptibility to deformation‐induced DNA damage appeared to be cell type specific. It also did not seem to correlate with variances in nuclear deformability or lamin levels but rather with mutations in the gene encoding p53, already known for facilitating the restart of DNA replication at stalled forks.^185^

Cancer cell genomic stability, or the lack of it, can also depend on the interaction of lamin A with other proteins. In ovarian cancer cells, lamin A knockdown resulted in an abnormal accumulation of the Heat Shock Protein 90 (HSP90).^186^ Considering the crucial role of HSP90 in interacting with DNA repair‐associated proteins,^187^, ^188^ interfering with its nucleocytoplasmic transport could ultimately compromise DNA repair mechanisms. Lamin A/C also influences the localization of AKTIP, a tumor‐associated factor important for telomere integrity and genomic stability.^189^, ^190^ The analysis of AKTIP localization patterns across three cancer cell models revealed colocalization with lamin A/C at the NM.^191^ Accordingly, breast cancer cells, which had lower levels of lamin A, showed reduced AKTIP at the NM compared with other cell lines. Various nuclear morphometric parameters—such as circularity, solidity, roundness, and nuclear bleb frequency—were also assessed and condensed into a single morphometric index termed *nuclear aberration*. The integration of expression levels and nuclear aberration data revealed an inverse correlation between these parameters and the positioning of AKTIP at the NM. In addition to regulating protein localization, lamin A could affect intranuclear chromatin localization. For example, upon TME stiffness increase and cell spreading, the epitope in the Ig‐domain of lamin A was masked, impacting lamin‐chromatin interactions.^62^

Overall, these studies highlight the complexity of lamin A/C's role in cancer and how various factors intersect to influence its expression levels across different cancer types. Once considered merely a structural component, lamin A/C is now emerging as a pivotal contributor to the onset and progression of various tumors. Organization and levels of lamin A/C are both dynamically regulated in response to the physical ME and can modulate cellular mechanotransduction signalling.

### 3.3 Lamin B1 and B2

B‐type lamins have garnered attention in cancer research due to their dysregulation across various cancer types. However, the implications of this dysregulation remain a topic of debate, with conflicting results reported by different studies. *LMNB1* upregulation in hepatocellular carcinoma, ovarian, pancreatic, liver, renal, and gastric cancers correlated with poor prognosis. Conversely, in lung and breast cancers, downmodulation of *LMNB1* has been associated with increased aggressiveness.^121^, ^192^, ^193^ On the other hand, *LMNB2* overexpression enhanced migration, cell proliferation, and cell cycle progression in bladder, colon, and non‐small cell lung cancers.^118^, ^124^, ^130^ Despite these observations, the mechanistic underpinnings of lamin B protein dysregulation in cancer progression remain poorly understood. Recent research has shed light on a potential mechanism demonstrating that lamin B1 overexpression in normal fibroblasts induces telomeric fusions and telomere losses. This telomeric instability occurs through disruption of the shelterin complex, a safeguard of telomere integrity.^194^ Overexpression of lamin B1 in cancer cells could similarly induce telomere dysfunction, exacerbating chromosomal instability during tumorigenesis. A role for lamin B1 in regulating the epigenetic mechanisms of tumor cells has been elucidated in lung cancer. The depletion of lamin B1 induced significant alterations in the H3K27me3 landscape, revealing a connection between both aberrant nuclear architecture and epigenetic patterning and the aggressiveness of lung cancer.^117^ Specifically, the loss of lamin B1 reactivated the proto‐oncogene RET, thus facilitating EMT, tumor proliferation, and enhanced cell migration in lung cancer cell lines. Even the loss of a single lamin B1 allele appeared sufficient to trigger spontaneous lung tumor formation.^117^ On the other hand, TGFβ‐ or SNAIL‐induced EMT in breast cancer cells induces cytokinesis failure, leading to increased nuclear flexibility and deformability. These events necessitate concomitant cellular proliferation during EMT, and are strictly correlated with alterations of NE proteins including lamin B1.^195^ Investigations into myelodysplastic syndromes and acute myeloid leukemia (AML) have unveiled a notable association between lamin B1 and chromosomal aberrations. The loss of the long arm of chromosome 5 (5q), which encompasses the *LMNB1* gene, emerges as the most frequent chromosomal deletion observed in patients with these malignancies.^196^ A recent study underscored the significance of *LMNB1* loss in hematopoietic stem cell function during malignant transformation. *LMNB1* deficiency induced nuclear aberrations and functional impairments in hematopoietic stem cells, disrupting 3D genome organization and leading to the dysregulation of lineage‐specific transcription factors, thereby impeding hematopoiesis. Moreover, 5q loss was linked to compromised DNA repair efficiency and portended a poorer prognosis for AML patients.^129^

Lamin B1 reduction also contributes to a newly observed bidirectional mechanical feedback pathway, where chromatin alterations are influenced by external mechanical signals and, in turn, affect nuclear and cellular mechanics. NE rupture under high levels of mechanical stress can expose DNA into the cytoplasm, activating the cyclic GMP–AMP synthase (cGAS)–STING (stimulator of interferon genes) pathway. Upon viral infection, cGAS acts as a cytosolic DNA sensor, binding to cytoplasmic double‐stranded DNA and generating 2′3′‐cyclic‐GMP–AMP, which then activates STING. This leads to the transcription of inflammatory response genes and activation of innate immune responses.^197^ This antiviral immunity mechanism, however, can have both anti‐ and pro‐tumorigenic functions. It aids in eliminating precancerous cells through autophagy and permanent cell cycle arrest, thus promoting senescence.^198^, ^199^ Conversely, highly genetically unstable cancer cells can activate a STING‐dependent noncanonical pathway that prompts NF‐kB activation and inflammatory response, ultimately fostering invasion and metastasis.^200^ Recent research reported a new mechanism for cGAS‐STING pathway activation upon NE ruptures due to chronic strains and stresses associated with unjamming.^201^ Cellular unjamming, a newly identified process characterized by collective and cooperative cellular motion resembling fluid flow, facilitates the acquisition of migratory behavior by both normal and cancerous cells.^202^, ^203^ For malignancy to arise, a certain level of fluidity is necessary to enable tissue proliferation, migration, and dissemination. The transition from a solid or jammed state to a liquid‐like or unjammed state at the tissue level could be a complementary or alternative mechanism for cell invasion. This transition correlates with the upregulation of the endocytic, promigratory gene *RAB5A*, driving a shift towards collective motion. This latest study found that the mechanical deformations experienced by cells and nuclei during unjamming induced a cellular mechano‐protective mechanism involving nuclear size and rigidity changes, heterochromatin redistribution, and perinuclear actin architecture remodeling.^201^ Moreover, the expression of RAB5A correlated with reduced levels of lamin B1 (but not lamin A/C) mRNA and protein, potentially compromising nuclear integrity and activating cGAS. Chronic mechanical stress and lamin B1 reduction in RAB5A‐fluidized monolayers could have also elicited genome‐wide structural alterations. However, while H3K27me3 heterochromatin slightly increased, constitutive H3K9me3 – normally associated with the lamina and potentially used as a mechanism to dissipate forces—displayed no changes.^101^, ^201^ Thus, RAB5A‐fluidized monolayers seemed to mount a complex mechanoprotective response, which leads to decreased nuclear plasticity and softness, suggesting the possibility that these monolayers are less capable of dissipating mechanical energy to prevent DNA damage.^101^ Indeed, as previously discussed, epigenetic changes in chromatin configuration can directly impact the physical properties of the nucleus, with higher levels of heterochromatin increasing nuclear stiffness and elevated euchromatin enhancing nuclear deformability.^107^, ^204^ Since chromatin is tethered to both the lamina and the INM via LADs,^81^ it is not unexpected that changes in NE components observed in cancer cells may correlate with chromatin modifications and vice versa.

### 3.4 Chromatin organization and LADs

3D genome organization, spatial chromatin rearrangements, and consequent epigenetic reprogramming are well‐known for regulating critical biological processes that are receiving increasing attention in the context of cancer.^205^, ^206^

The maintenance of chromatin compartmentalization is pivotal during constricted cell migration. For instance, increasing euchromatin or reducing heterochromatin hinders the migration of melanoma and fibrosarcoma cells.^207^, ^208^, ^209^, ^210^ Alterations in heterochromatin condensation also occur during cellular reorganization such as during EMT.^211^, ^212^ Moreover, chromatin decondensation can promote the invasion of breast cancer cells into dense ECMs and suppress invasion into less compact matrices.^110^ Conversely, external mechanical forces can influence nuclear mechanics and chromosome structure. Mechanical signals from the cell's surroundings can cause chromatin to redistribute spatially,^213^ and external cellular forces can even impact gene expression by directly deforming chromatin.^105^ Of note, the process of constrained migration itself might induce changes in chromatin modifications.^214^ Specifically, confined 3D migration within microfluidic devices increased the heterochromatin marks H3K9me3 and H3K27me3 in cancer cells and fibroblasts for days. Heterochromatin formation intensified with the degree of confinement, relied on histone‐modifying enzymes, and was affected by NE proteins and stretch‐sensitive ion channels. Despite the correlations observed between chromatin structure and nucleus deformation and mechanics, there remains a gap in understanding whether the 3D organization of the genome influences or is influenced by constricted migration. In other words, the modified expression of NE proteins identified in different cancers could directly impact chromatin organization and gene expression. But it is also plausible that alterations in the expression of NE proteins resulted from, rather than caused, changes in chromatin organization. Recent experiments and modeling studies suggested that differences in the 3D genome and nucleus may arise from a combination of selection within an initially heterogeneous cell population and changes induced by constricted migration.^215^ Invasive human melanoma cells that had migrated repeatedly through tight constrictions exhibited enhanced migration efficiency and distinct phenotypes compared with cells that had not passed through such constraints. These cells had specific and stable discrepancies in 3D genome organization, particularly evident in A/B compartmentalization, lamin A/C localization, and gene expression patterns. Interestingly, some of these structural changes also appeared in breast cancer cells following constricted migration.^215^

A novel study revealed that the loss of 3D genome organization, with changes involving both A‐ and B‐compartments, is related to breast cancer progression contributing to the loss of gene expression control.^216^

Moreover, increasing evidence indicates the nuclear periphery as a dynamic compartment involved in cancer epigenetic reprogramming that, as described in senescence, mainly occurs in domains coinciding with LADs.^206^, ^217^ Indeed, LADs are hypomethylated across different cancer types,^218^, ^219^, ^220^ and subjected to higher ultraviolet‐light‐induced damage respect to non‐LAD regions.^221^ A very recent study identified specific genes (nucleoporin *(NUP)98* and methyl‐CpG binding protein *(MECP)2*) in LAD domains as potential drivers in AML.^222^

The alterations of NE composition can also predict impairments in LADs‐nuclear lamina contacts with possible consequences in cancer progression. Indeed, LADs spatial rearrangement due to changes in the nuclear lamina could potentially relocate oncogenes away from the lamina, resulting in their expression as it happens during cellular differentiation.^223^, ^224^, ^225^ It is becoming evident that coordinated spatial positioning of LADs and their interaction with NE components play a fundamental role not only in cellular differentiation but also in diseases. The importance of NE proteins in LADs regulation is exemplified in Hutchinson‐Gilford progeria syndrome (HGPS), a progeroid disease caused by a mutant lamin A precursor (named progerin).^226^ Indeed, HGPS patients exhibit a strong deregulation of LADs which appear to undergo substantial relaxation, thus contributing to disease‐specific gene expression.^227^ Recent evidence on tumor models shows that lamin B1 is essential for tethering LADs at the nuclear lamina in breast cancer cells.^228^ When absent, a portion of LADs detached from the lamina and relocated to the nuclear interior, resulting in chromatin relaxation and genomic material redistribution. The use of the clustered regularly interspaced short palindromic repeats (CRISPR)‐SunTag system to label genomic loci in living cells further revealed that the movement of genomic loci is more pronounced in lamin B1‐depleted cells, highlighting the dependence on chromatin decompaction. By combining imaging and sequencing techniques, lamin B1 (and its interaction with the nuclear lamina) emerged as a critical element for maintaining LADs, compacting chromatin, organizing chromosome territories, and delineating A/B compartments in human breast cancer cells.^228^ Lamin B1 can also interact with euchromatic LADs (eLADs) during EMT.^229^ Throughout the EMT process, there was a notable rise in the formation of newly created eLADs in the B compartment, accompanied by increased lamin B1 levels in normal mouse epithelial cells. Genes residing in eLADs must revert to a silent state upon cells acquiring the mesenchymal phenotype, highlighting the importance of LAD positioning within the nucleus.^229^ While further studies are needed to elucidate the mechanisms in human cells, it is plausible that alterations in lamin B levels in cancer cells could impair EMT. The interplay between NE proteins and LADs organization goes beyond lamins. For instance, the LBR plays a crucial role in anchoring specific chromatin regions to the nuclear periphery,^205^, ^230^ which typically acts as a transcriptionally repressive environment.^231^ LBR interacts also with HP1 and histones H3/H4.^232^ Moreover, LBR disruption could control the development of drug resistance by affecting 3D genome organization.^233^ Extensive research has shown that drug resistance to the chemotherapy drug Taxol depends on the transcriptional activation of *ABCB1* (ATP binding cassette subfamily B member 1), the gene coding for the P‐glycoprotein (P‐gP) drug‐efflux pump.^234^, ^235^ In immortalized human retinal pigment epithelial cells, this activation required the detachment of the *ABCB1* locus from the nuclear lamina. Only the depletion of *LBR*, but not lamin A/C or B, could render the locus permissive to gene activation.^233^ This model, where the detachment of a repressed gene from the nuclear lamina triggers its activation, could have significant implications for understanding acquired drug resistance to chemotherapeutic drugs. Another NE protein, emerin, has the potential to influence chromatin organization, as it directly interacts with chromatin modifiers and transcriptional repressors such as the death‐promoting factor Btf, the splicing‐associated factor YT521‐B, and the transcriptional repressor GCL (germ cell‐less).^236^, ^237^, ^238^ In the nucleus of colorectal adenocarcinoma cells, emerin modulated the spatial organization of chromosome territories by functioning as a mechanotransducer.^239^ Specifically, exposing the cells to decreased matrix stiffness triggered emerin phosphorylation, the misplacement of NE proteins within the nucleoplasm, and the displacement of chromosome territories 18 and 19. In this context, emerin phosphorylation could act as a regulatory factor upstream of lamin localization, as the phosphorylation of emerin at the Tyr99 residue corresponded to the interaction domain between emerin and lamin A/C.^239^ The substrate stiffness‐dependent phosphorylation of emerin by Src kinase could thus disrupt the emerin–lamin A/C interaction, leading to the misplacement of lamin A into the nucleoplasm and a portion of emerin outside the nucleus.^240^

### 3.5 Emerin

Emerin plays a role also in determining nuclear size, shape, and polarity, binding to lamins at the NE or being sequestered in the ER upon lamin loss.^241^, ^242^, ^243^ In cancer contexts, the *EMD* gene presents over a hundred mutations associated with malignancy, many of which influence emerin expression and folding.^114^ Recently, a study unveiled a significant association between emerin expression in invasive breast cancer tumor tissue and survival time.^114^ Specifically, a reduction of emerin by 40–50% in invasive breast cancer cells compared with control cells led to structural abnormalities in the nucleus previously associated with increased cell migration, intravasation, and extravasation. The study also reported that overexpression of emerin effectively counteracted nuclear irregularities and inhibited metastatic tendencies both in vitro and in vivo. This effect was contingent upon emerin's ability to bind to the nucleoskeleton. These findings support a model wherein emerin's interaction with the nucleoskeleton governs nuclear architecture, thereby influencing the metastatic potential of breast cancer cells. In line with these data, previous research demonstrated that emerin depletion or misplacement resulted in nuclear shape instability linked to increased metastasis formation in prostate cancer in vivo models.^244^ The induced nuclear shape instability mirrored the effects seen upon depletion of lamin A/C or Diaphanous‐related formin 3 (DIAPH3/mDia2), a cytoskeletal protein often absent in metastatic breast and prostate cancer.^245^ Cancer cells lacking DIAPH3 displayed an amoeboid phenotype, characterized by rounded cell morphology, high actomyosin contractility, and rapid extension and retraction of membrane protrusions, which allow cells to move rapidly through ECMs.^246^ As for nuclear instability, the nuclei of DIAPH3‐deficient cancer cells presented NE disruption, NM blebbing, cytosolic fragments containing nuclear material, and the release of extracellular vesicles with nuclear content. Emerin depletion induced similar nuclear alterations and enhanced metastasis in murine models, suggesting that various molecular mechanisms converge toward a phenotype characterized by amoeboid behavior and increased aggressiveness.^244^ Emerin is also found in micronuclei present in prostate cancer cells, with its subsequent loss at the NE. The loss of emerin localization leads to a more invasive phenotype, which is associated with a worse prognosis.^247^ Consistently, a different study found that enhanced emerin expression increased nuclear stiffness in melanoma cells.^248^ In this model, emerin's activity appeared finely tuned to facilitate ameboid migration, as both up‐ and down‐regulation of its levels led to a decreased proportion of mobile cells and reduced cell velocity. Furthermore, overexpression of emerin significantly reduced the area of plasma membrane blebs, the initial step in ameboid movement. Isolated nuclei overexpressing emerin were stiffer than those of cells with intermediate emerin levels, and emerin‐mediated effects relied on its phosphorylation by the tyrosine‐protein kinase Src. Upon removal of Src phosphorylation sites, the overexpression of emerin alone failed to hinder rapid ameboid migration. Alterations in nuclear stiffness, leader bleb area, and subsequent melanoma migration were contingent on emerin being correctly localized at the INM.

Despite the aforementioned observations, the role of emerin in nuclear softening or stiffening in response to changes in TME mechanics remains unclear. Emerin could act as a mechanosensor, modulating chromosome territory positions and gene expression in response to changes in ECM mechanics.^239^, ^249^ Additionally, emerin regulates actin polymerization and interacts with the SUN‐domain proteins of the LINC complex, which transduce mechanical force to the nucleus.^250^, ^251^ However, the mechanisms by which emerin senses these mechanical signals and how its reduction or mutation leads to dysfunctional mechanotransduction in cancer remain unclear. Cells lacking emerin exhibited nuclear morphology abnormalities, leading to heightened nuclear deformability and impaired mechanotransduction.^58^ Proper emerin distribution within the INM is crucial for connecting the nucleoskeleton and cytoskeleton, thereby enhancing stability during nuclear deformation.^252^ Additionally, emerin interacts with nuclear actin and nuclear myosin I, aiding in the formation of a cortical nuclear actin‐myosin network around the NE, which in turn reinforces NE structural integrity.^250^ Reduced emerin levels could thus compromise the stability of cancer cell nuclei, making them more susceptible to deformations and ultimately increasing cell migratory capacity. Moreover, emerin interacts with proteins involved in different signaling pathways that are frequently dysregulated in cancers (e.g., Notch, TGF‐β, Wnt, MAPK, IGF).^114^ Yet, how emerin loss or mutation contributes to the disruption of these crucial pathways in cancer cells remains unclear.

Emerin dysregulation may also contribute to DNA instability, a common trait of tumor cells. By interacting with various transcription regulators, emerin controls the expression of numerous genes and the correct reassembly of the nucleus after mitosis.^241^, ^253^ During mitosis, emerin relocates to the centrosomes and microtubules, moreover the deletion of the *EMD* gene leads to abnormal, prolonged mitosis.^254^, ^255^ Cells derived from patients with Emery–Dreifuss muscular dystrophy type 1, which lack emerin expression, showed delayed cell cycle progression and impaired DNA damage repair under oxidative stress.^256^ Emerin is indeed known to interact with DNA repair proteins, and its knockout in the model organism *C. elegans* is associated with hypersensitivity to DNA damage.^241^, ^257^ Among emerin binding partners are several factors involved in epigenetic regulation and the histone modification machinery. For example, it directly interacts with HDAC3 and enhances its catalytic activity, thus facilitating LAD localization to the NE and their transcriptional repression.^258^ During myogenic differentiation, emerin binds to methyltransferases and recruits EZH2, an enzymatic catalytic subunit of PRC2 that can alter downstream target gene expression by forming H3K27me3.^259^ These findings suggest a potential mechanism for initiating and maintaining the repression of genes at the nuclear periphery, in which emerin may be crucial in maintaining histone methylation homeostasis. Cancer cells with a delocalized or reduced emerin expression could thus fail to properly control the organization of repressive chromatin, resulting in aberrant gene regulation.

### 3.6 LINC complex

In recent years, studies have shown the presence of deregulated expression of SUN proteins in various types of malignant transformations. SUN2 can function as a tumor suppressor by inhibiting cell proliferation in embryonal cancers of the central nervous system and inhibiting cell migration of lung cancer cells.^119^, ^260^ On the other hand, overexpression of SUN4 seemed to support the migration of lung cancer cells and contributed to the progression of hepatocellular carcinoma by impacting lipid metabolism.^261^, ^262^ A recent study delved into the mechanism that governs SUN2 turnover, shedding light on how the buildup of nondegradable forms of SUN2 led to the disruption of nuclear architecture in Hela cells and impeded the repair of DSBs (double‐strand breaks) in osteosarcoma cells exposed to ionizing radiation.^263^ SUN2 homeostasis thus appeared critical for cancer progression, as the heightened degradation of SUN2 via ubiquitination led to increased proliferation of ovarian cells and inhibited apoptosis.^264^ SUN2 has emerged as a novel interacting protein of the spliceosome complex. Knockdown of SUN2 in invasive breast cancer cells impaired the splicing of sororin, which stabilizes sister chromatids during DNA replication. As a result, cell proliferation was impaired.^265^ SUN1 and SUN2 were recently identified as crucial mediators in remodeling the nucleus of macrophages during M1 polarization in response to inflammatory signals—a tightly regulated process whose imbalance often correlates with tumor progression. Depletion of SUN1/2 reduced the size and stiffness of the nuclei of murine macrophages, leading to alterations in chromatin conformation and the expression of M1 macrophage genes. As a result, nuclear softening facilitated the migration of M1 macrophages into surrounding tissues, where they produced proinflammatory cytokines.^266^ Manipulating these proteins could thus be a means to enhance the antitumor activity of immune cells or counteract the immunosuppressive strategies employed by tumor cells.

Changes in the levels of SUN proteins, particularly SUN1 and SUN2, also influence cancer cell migration, with tumor type‐dependent effects. For instance, primary colorectal tissues and distant metastases presented lower levels of SUN2 compared with normal colon tissue, with higher SUN2 expression correlating with favorable overall survival.^125^ In vitro and in vivo experiments demonstrated that overexpression or knockdown of SUN2 respectively hindered or facilitated cell migration and invasion. In this context, SUN2 acts as a tumor suppressor by downregulating the expression of brain‐derived neurotrophic factor (BDNF), thus inhibiting the BDNF/tropomyosin‐related kinase B signaling whose overexpression seemed involved in several carcinogenic processes.^125^ Conversely, the invasiveness capacity of breast cancer cells depleted of SUN1 was lower than control cells.^267^ However, the 10 splicing variants identified for the human SUN1 gene seemed to have both overlapping and distinct roles in controlling cell migration, hinting at the existence of varied LINC complexes with specific functions. SUN1 and SUN2 are mechanosensory factors that restrain cancer cell invasion upon mechanical signals introduced as low‐intensity vibration (LIV).^268^ LIV is a low‐magnitude, high‐frequency vibration that, in this context, was meant to mimic physical exercise. Applied to human breast cancer cells, LIV decreased matrix invasion and impaired secretion of osteolytic factors in a SUN1‐ and SUN2‐dependent manner. On the other hand, another study demonstrated that low‐dose X‐irradiations in human breast cancer cells led to up‐regulation of SUN1 expression, which in turn played a central role in the EMT events through the Wnt/β‐catenin signaling pathway.^269^

Over the past decade, numerous studies have highlighted a connection between the LINC complex and focal adhesions (FAs). The latter are clusters of transmembrane and scaffolding proteins—including integrins, vinculin, and zyxin—that bridge ECM and the contractile actin.^270^ FAs interact with the LINC complex through direct links between the actin cytoskeleton and nesprins, facilitating the transmission of mechanical signals from the ECM to the nucleus.^73^, ^271^, ^272^, ^273^ In turn, perturbation of the LINC complex can influence the maturation of FAs, revealing a bidirectional exchange of signals between these two protein assemblies.^274^, ^275^, ^276^, ^277^ It was recently shown that SUN1 depletion led to diminished activation of integrin β1 and reduced recruitment of vinculin and zyxin to nascent FAs, both known to rely on traction force.^278^ Hence, the physical tethering of the nucleus to the cytoskeleton via SUN1 emerges as critical for proper actin arrangement, intracellular traction force generation, and FA maturation, all essential for facilitating cell migration. Deciphered mechanisms underlying the bidirectional mechanical feedback between ECM and nucleus are few and far between. A notable example delved into the function of SUN1 in controlling cancer cells’ ability to remodel ECM barriers.^279^ The invasion of carcinoma cells is fueled by the enzymatic breakdown of the pericellular matrix orchestrated by membrane‐type 1 matrix metalloproteinase (MT1‐MMP).^280^, ^281^ Cancer cells tune their surface‐exposed MT1‐MMP levels through trafficking from storage compartments within late endosomes/lysosomes.^281^ A study recently reported that cell invasion through tight pores of a collagen meshwork triggered a response where MT1‐MMP‐positive storage endosomes became polarized in front of the nucleus. The nucleus–centrosome connection via nesprin‐2 and the dynein adaptor Lis1 was crucial for this response. Based on these findings, a model was proposed where nuclear movement through tight spaces operates on a digest‐on‐demand basis. Here, confined migration prompts the polarization of MT1‐MMP storage compartments and the breakdown of ECM proteins ahead of the nucleus depending on a LINC‐mediated nucleus‐microtubule linkage.^280^ Besides ECM degradation, cancer cells navigate through exceptionally narrow constrictions by softening their nuclei, as discussed before.^22^, ^134^ However, a certain degree of NE stiffness is required to prevent severe NE ruptures.^148^ In this context, the ataxia telangiectasia and Rad3‐related protein (ATR) behaved as a mechanotransduction element that ensured proper mechanical coupling between the cytoskeleton and the NE.^282^ During migration through narrow constrictions, the LINC complexes at the leading edge of the nucleus tightly bound to the cytoskeleton, leading to sporadic ruptures at the ONMs, while the NE at the rear part of the nucleus remained intact. In other words, the nucleus became polarized at the NE level. Cells lacking ATR, however, failed to maintain the coupling between nesprin‐2 and the cytoskeleton and to adequately respond to mechanical stress at the leading edge of the nucleus, accumulating NE invaginations and extensive ruptures in both NMs. Furthermore, these NE invaginations, coupled with partially condensed chromatin, may hinder efficient nuclear squeezing, and impede the effective repair of NMs. ATR depletion thus impaired the 3D invasion and lung homing of cancer cells, revealing a potentially critical role in metastasis. Since ATR was previously involved in the activation of the DNA damage checkpoint, the protein seems to have two opposite roles in cancer progression.^283^

As for nesprins, the understanding of their contribution to tumorigenesis is still somewhat limited. Recent studies have suggested potential roles in DNA damage response, genome organization, transcriptional regulation, and cell migration.^284^, ^285^, ^286^ Moreover, bioinformatic analyses have unveiled a prognostic potential for *SYNE3* expression (encoding for nesprin‐3), revealing that higher *SYNE3* levels correlate with extended overall survival in patients with different types of tumors.^287^ While these findings offer promising insights into the nesprin family's role in cancer, further experimental validation is needed.

## 4 THE ROAD TO CLINICAL APPLICATIONS

In this section, we will discuss how existing drugs could be repurposed to target nuclear function and structure, opening new therapeutic options. We will also explore recent advances in AI‐driven NE analysis, showing how these technologies could use nuclear alterations as potential biomarkers for diagnosing or predicting outcomes in specific tumor subtypes.

### 4.1 Targeting the NE

Alterations in nuclear morphology and mechanics correlate to enhanced metastatic potential in various cancers.^288^ Recently, the treatment potential of targeting nuclear size changes to curb cell migration has been investigated.^289^ Some compounds approved by the Food and Drug Administration and the European Medicines Agency were screened to identify a subset of drugs capable of reversing nuclear size alterations, specifically tailored to individual tumors. These compounds also reduced cell migration and invasion.^289^ With their potential for innovative combinatorial therapies targeting nuclear morphology, these repurposed drugs highlight the cell nucleus as a druggable target for cancer treatment. In that context, the most reliable and consistent molecular effector of nuclear‐size rectifier drugs was identified in lamin A.^288^, ^289^ This is not unexpected as lamin A is downregulated in a group of tumors and upregulated in another group and changes in its levels correlate with cancer severity. Our hypothesis based on our recent research is related to the reported data by Discher and collaborators showing that each original untransformed cell type holds a fixed level of lamin A depending on the stiffness of its own tissue. In fact, hard tissues express high *LMNA* transcript levels, while in soft tissues like brain very low lamin A amount is detected.^74^ Based on this knowledge, changes in lamin A levels might withdraw the cell from the tissue‐specific program of differentiation and function and redirect cell behavior towards a more undifferentiated phenotype. Thus, upregulation or downregulation of lamin A in transformed cells could depend on the original condition. Along this line, osteoblasts express higher levels of lamin A under physiological conditions than in bone cancers.^144^ Conversely, brain tumor cells express higher lamin A levels upon malignant transformation.^146^

To date, there are no anticancer drugs specifically targeting NE components. However, several compounds can modulate lamin A levels. The regulation of lamin A in cells occurs both transcriptionally and post‐translationally, either by modulating the processing rate of prelamin A or by altering the phosporylation of lamin A. Drugs that affect *LMNA* gene expression, such as ATM inhibitors, or those that impair prelamin A processing, such as statins (which impact prelamin A farnesylation), or Akt inhibitors (which reduce lamin A Ser22 phosphorylation required for nuclear lamina disassembly), have proven effective in reducing cancer cell proliferation, migration, and overall tumor progression.^149^, ^159^, ^160^, ^288^, ^289^ Of note, some of these drugs simultaneously restore lamin A levels and correct the aberrant nuclear shape seen in tumor cells.^288^, ^289^ Studies on laminopathic cells have shown that prelamin A levels, post‐translational modifications, and/or mature lamin A amounts play a critical role in nuclear remodeling and structural maintenance, which, in turn, impact cell fate.^76^ Beyond their effects on nuclear morphology, drugs that regulate lamins can restore their interaction with key cancer‐related molecules, such as 53BP1. This protein is recruited by prelamin A and reduced in cells that accumulate mutant prelamin A. Besides, ATM is impaired by prelamin A accumulation, and its inhibition leads to lower lamin A levels, impacting nuclear mechanosensing and chromatin remodeling.^76^, ^160^, ^290^, ^291^, ^292^ Moreover, treatment with HDAC inhibitors has been shown to restore lamin A/C functionality in progeroid cells.^293^ These observations, along with the growing list of molecules acting directly or indirectly on the NE (reviewed in Refs. ^288^ and ^289^), have important therapeutic implications for cancer, which, in our view, have been largely overlooked or underestimated. For instance, recent studies suggest that statins, which target prelamin A processing and lamin A transcriptional regulation,^149^ may not only have therapeutic benefits but could also serve as a preventive measure against breast tumors. However, careful consideration must be given to the type of molecule used and its target tissues.^294^

Many small molecules alter lamin A expression and its nuclear recruitment, and these compounds have been long used in chemotherapy and other cancer treatments. 5‐Azacytidine, a DNA methylation inhibitor widely used in blood cancer chemotherapy, increases *LMNA* gene expression and lamin A/C levels. In Ewing Sarcoma, this drug raises lamin A levels and reduces cellular migration.^149^ It would be interesting to explore whether there is a link between 5‐azacytidine's effectiveness as an antitumor agent and lamin A/C expression levels across different cancers. Another frequently used antitumor drug, lonafarnib, targets the enzyme farnesyltransferase and, like statins, inhibits the farnesylations of prelamin A (and prelamin B).^76^ While its ability to impair KRAS farnesylation and activity has shown limited success as an anticancer agent, recent studies combining lonafarnib with KRAS‐G12C inhibitors have revealed a synergistic effect, likely due to their combined impact on prelamin A processing.^295^ Finally, trichostatin A, a histone deacetylase inhibitor, interferes with normal and pathological lamin A interactions and affects heterochromatin organization, potentially rescuing lost heterochromatin domains in progeria. It is now becoming a well‐accepted therapeutic agent in breast cancer.^293^, ^296^ In all of these cases, understanding the role of lamin A expression levels and intermolecular interactions is crucial and warrants further investigation.

### 4.2 Novel technologies

Alterations in nuclear morphology are valuable indicators of malignant transformation.^297^, ^298^ Recent advances in deep learning techniques have brought forth promising avenues in oncology, harnessing the potential of multi‐layer neural network algorithms inspired by the neurological architecture of the human brain.^299^ Among these techniques, convolutional neural networks (CNNs) stand out, applying small feature‐filter matrices to extract granular features from image data for predictive purposes.^300^, ^301^ CNNs could effectively identify critical cell regions and extract details from high‐resolution histology images of breast, prostate, colon cancers, and lymphoma,^299^ with an interpretation accuracy that equaled that of anatomical pathologists. However, the deep learning model's effectiveness hinges on ample, meticulously processed training data, which poses a challenge for robust, generalizable models.^302^ Overcoming these constraints is vital for the widespread adoption of deep learning techniques in oncological diagnostics.

Cancer is a multifaceted disease with varied microscopic, macroscopic, and molecular features affecting patient outcomes. Integrating histopathological images with clinical data and omics analyses could thus anticipate patient prognosis and clinical response, and pinpoint specific cancer signatures. A recent study introduced Ceograph, a novel cell spatial organization‐based graph convolutional network designed for analyzing cell spatial arrangement from pathology images.^303^ Ceograph incorporates nucleus morphology features to transform tissue images into spatial maps of cells, enabling assessment of the relationship between cell morphology, spatial interactions, diagnosis, and clinical outcomes. Ceograph proved useful in predicting malignant transformation risk in individuals with oral disorders and assessing treatment response in lung cancer patients.^303^ Soon, this deep learning approach will also hopefully provide insights into the role of the cell nucleus in mediating treatment resistance mechanisms.

The ever‐increasing understanding of nuclear mechanics and mechanotransduction has spurred the development of a so‐called mechanotherapy. This approach uses mechanical stimuli to selectively target tumor cells by exploiting the different mechanical properties of malignant and healthy cells.^304^ Various techniques, such as ultrasound irradiation, mechanical stretches, and shock waves, have shown promise in laboratory and animal studies (reviewed in Ref. 304). Among these methods, low‐intensity pulsed ultrasound (LIPU) increased cancer cell death rates in breast cancer and melanoma.^305^ High‐frequency LIPU proved effective in restraining cervical cancer growth and enhancing overall survival in tumor‐bearing mice. LIPU worked by triggering the intracellular explosion of folate‐conjugated nanobubbles, which selectively eliminate cancer cells.^306^ Ultrasound irradiation could thus represent a promising approach for selectively targeting cancerous nuclei. Of note, its lower side effects make low‐intensity LIPU a potentially safer option for cancer therapy than high‐intensity ultrasound.^307^

While mechanotherapy does not employ novel techniques, the growing recognition of nuclear mechanics’ significance in cancer development has been facilitated by advancements in high‐precision measurement technologies for assessing nuclear mechanical properties.^42^, ^304^, ^308^, ^309^ Numerous technological tools exist to characterize and manipulate the mechanical characteristics of the cell nucleus, as extensively reviewed in Refs. ^42^ and ^310^. Nuclear mechanics such as elasticity, viscosity, or stiffness can be quantified using diverse techniques. Among these, atomic force microscopy employs flexible cantilevers to apply mechanical forces to the nucleus while monitoring sample deformations.^310^, ^311^ Micropipette aspiration represents another approach, assessing the mechanical properties of both the nucleus and the entire cell by applying negative pressure to the samples and measuring resultant deformations.^312^, ^313^ Additionally, numerous studies have utilized Förster resonance energy transfer‐based biosensors to analyze forces acting on the nucleus through the integration of these sensors into NE components such as nesprins.^314^, ^315^ In vitro manipulation of nuclear mechanics can mimic the diverse mechanical forces that cells encounter in vivo.^42^, ^310^ To simulate physiological or pathological conditions, for instance, the nucleus can be compressed using 3D cell confinement platforms or stretched by applying specific forces.^316^, ^317^, ^318^ Microfluidic devices are valuable tools for investigating how mechanical forces influence cell migration. These tools mimic characteristics of the cellular ME, featuring varying pore sizes to exert stress on the nucleus as cells pass through, thereby enabling the characterization of nuclear deformation during migration processes.^42^, ^319^ Although limited to preclinical data, these techniques can allow the establishment of comprehensive mechanical models of cancer cell nuclei. Such models could then serve as a foundation for exploring novel therapeutic approaches to complement traditional anticancer strategies.

## 5 CONCLUSION AND PROSPECTS

The widespread occurrence of NE alterations in malignant cells is particularly striking, as nuclear abnormalities are pervasive across various cancer types, irrespective of their tissue origins, mutational profiles, or signaling dependencies. Such frequent changes strongly imply that alterations in nuclear structure play a crucial and active role in cellular transformation. This review highlights how the NE, once considered a mere structural boundary, has emerged as a dynamic regulator of tumor progression, shaping the behavior of cancer cells. We explore how alterations in critical NE components—lamins, emerin, and LINC complex proteins—drive mechanotransduction, chromatin remodeling, gene regulation, and cell invasiveness. These changes not only reshape the physical structure of the nucleus but also fuel the malignant phenotype, empowering tumor cells with the ability to adapt, invade, and proliferate uncontrollably. However, only a few studies have successfully established direct links between the factors that initiate NE alterations, and the functional consequences observed in malignant cell behaviors. This highlights the urgent need to unravel the cause‐and‐effect relationships between changes in NE structure and composition and the tumorigenic process.

Currently, there is no clear, common mechanism to explain the deregulation of NE components in cancer progression, and few studies are actively addressing this gap. It remains unclear whether nuclear morphological changes simply correlate with other, more obscure cellular defects or whether they actively contribute to disease progression. Advancing our understanding of how the NE controls tumor progression holds promise for novel treatments that may revolutionize cancer care. Additionally, emerging technologies could soon harness nuclear alterations as powerful prognostic and diagnostic tools for specific tumor subtypes, offering transformative potential for cancer diagnosis and treatment.

## AUTHOR CONTRIBUTIONS

*Conceptualization, writing original draft*: F. P. *Review and writing*: A. P. *Images preparation*: S. T. *Review*: A. M. *Review*: C. P. *Review and writing*: G. L. *Conceptualization, supervision, review, and editing*: F. C. All authors have read and approved the manuscript for publication.

## CONFLICT OF INTEREST STATEMENT

The authors declare no conflicts of interest.

## ETHICS STATEMENT

Not applicable.

## Data Availability

Data sharing is not applicable to this article as no datasets were generated during the current study.
